# Zeolites with Divalent Ions as Carriers in the Delivery of Epigallocatechin Gallate

**DOI:** 10.1021/acsbiomaterials.3c00599

**Published:** 2023-08-04

**Authors:** Mariusz Sandomierski, Martyna Chojnacka, Maria Ratajczak, Adam Voelkel

**Affiliations:** † Institute of Chemical Technology and Engineering, Poznan University of Technology, ul. Berdychowo 4, 60-965 Poznań, Poland; ‡ Institute of Building Engineering, Poznan University of Technology, ul. Piotrowo 5, 60-965 Poznań, Poland

**Keywords:** zeolite, epigallocatechin gallate, drug delivery, controlled release

## Abstract

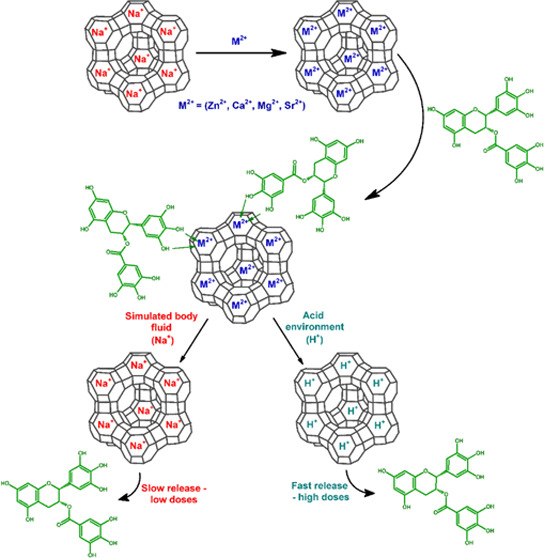

Epigallocatechin gallate (EGCG) is a compound with very high therapeutic potential in the treatment of osteoporosis and cancer. The disadvantages of this compound are its low stability and low bioavailability. Therefore, carriers for EGCG are sought to increase its use. In this work, new carriers are proposed, i.e., zeolites containing divalent ions of magnesium, calcium, strontium, and zinc in their structure. EGCG is retained on the carrier surface by strong interactions with divalent ions. Due to the presence of strong interactions, EGCG is released in a controlled manner from the carrier-ion-EGCG drug delivery system. The results obtained in this work confirm the effectiveness of the preparation of new carriers. EGCG is released from the carriers depending on the pH; hence, it can be used both in osteoporosis and in the treatment of cancer. The divalent ion used affects the sorption and release of the drug. The obtained results indicate the great potential of the proposed carriers and their advantage over the carriers described in the literature.

## 1 Introduction

Epigallocatechin gallate (EGCG) is a chemical compound derived from catechin. It is the most abundant polyphenolic component sourced from green tea extract.^1^ EGCG compound consists of eight free hydroxyl groups and three aromatic rings attached to a 5-member pyran ring. Due to its structure, EGCG shows biological activity which is associated with antioxidant, anti-inflammatory antidiabetic, antiobesity, and anticancer effects.^2^ Therefore, epigallocatechin gallate has significant potential in terms of treatment of various diseases such as cancers, osteoporosis, or neurodegenerative disorders.^3−5^

In recent years, particular interest has been brought to the anticancer properties of EGCG.^6^ According to previous studies, the antitumor mechanism of EGCG may include several pathways.^7^ First of all, epigallocatechin gallate can inhibit all processes involved in carcinogenesis—initiation, promotion, and progression.^8^ Furthermore, EGCG is also capable of inducing apoptosis of tumor cells and preventing angiogenesis, which leads to limiting the expansion of cancer.^9^ In comparison to other anticancer drugs, EGCG has several benefits. It is a nontoxic and naturally occurring compound.^10^ Besides, unlike the currently used angiogenesis inhibitors, it does not have to be administered intravenously or subcutaneously for long periods of time.^11^ In addition, research on EGCG has shown that it appears to target action against cancer cells without affecting healthy tissues.^12^

In the case of osteoporosis, delivering EGCG in small doses can improve bone mineral density, skeletal strength, and can also inhibit bone resorption.^13^ Additionally, the compound has the potential to promote osteoblastic activity, which can significantly contribute to reducing the negative effects of the disease.^5^

However, effective therapy with epigallocatechin gallate is still limited. One of the main problems during EGCG treatment is its poor bioavailability and stability.^1^ The catechin may easily oxidize in neutral or alkaline pH, undergoing degradation.^14^ Moreover, the hydroxyl groups present in the phenolic rings are susceptible to glucuronidation, methylation, and sulfonidation, which cause the loss of biological activity of the compound.^15^ Another problem limiting the use of EGCG in treatment is its low maximum concentration in human plasma after oral administration, which is about 0.32%.^16^ This value is far too low to ensure a proper efficiency. Moreover, EGCG is characterized by poor intestinal absorption, which is caused by oxidative decomposition at high temperatures and neutral or slightly alkaline conditions.^17^ Therefore, it is necessary to conduct research that will result in the development of new drug delivery systems that will increase the bioavailability and stability of the drug, thereby providing effective treatment.

Carriers for EGCG are most often based on nanostructures. They enabled high efficiency of drug loading and targeted transport of the compound, specific to the site of its release, which significantly improved the bioavailability of EGCG.^18^ One of the carriers used for delivering EGCG are lipids. Due to their high stability, biodegradability, and ability to controlled release, lipid-based nanocarriers are currently considered the most effective in delivering EGCG.^4,19^ Other EGCG delivery systems use chitosan. It may act as a surface modifier, a cellular uptake enhancer, and a carrier that releases the drug on demand. Moreover, chitosan nanoparticles can improve the bioavailability and intestinal absorption of EGCG.^1,20^ Different systems are based on liposomes, mesoporous silica, or gold nanoparticles, although new carriers that would be biocompatible, nontoxic, and would increase the stability of EGCG are still being sought.^1^ Good materials for this application may be zeolites. Zeolites are biocompatible, porous aluminosilicate materials with crystalline structure, consisting of 3D frameworks formed by [SiO_4_]^4–^ and [AlO_4_]^5–^, linked through oxygen atoms.^21^ They may be applied in industry as catalysts, adsorbents, or molecular sieves.^22^ They are also widely used in medicine and biotechnology. For example, they are components of scaffolds for bone tissue engineering, where they supply oxygen to the cells and stimulate the differentiation of osteogenic cells as well as inhibit bone resorption.^23^ One of the main fields where zeolites are used is drug delivery. In the past, they were used in the delivery systems of many anticancer drugs, such as 5-fluorouracil or mercaptopurine, as well as other drugs used to treat other diseases such as risedronate, ibuprofen, and indomethacin.^24−27^ The use of zeolites as drug carriers is possible mainly due to the regular and uniform shape of their pores, as well as their stability in the environment of body fluids, which has been proven, among others, for X type of zeolite.^28,29^ Moreover, the intracrystalline voids in the zeolite framework are occupied with water molecules and cations to balance the negative charge of AlO_4_.^30^ In natural zeolites, the cation could be, for example, Na^+^, Mg^2+^, or Ca^2+^, but also it could be exchanged for other metal cations such as Zn^2+^ or Sr^2+^.^31^ This property can be used to obtain zeolites with divalent ions to which the hydroxyl groups of the EGCG molecule could bind.^32^

**Figure 1:**
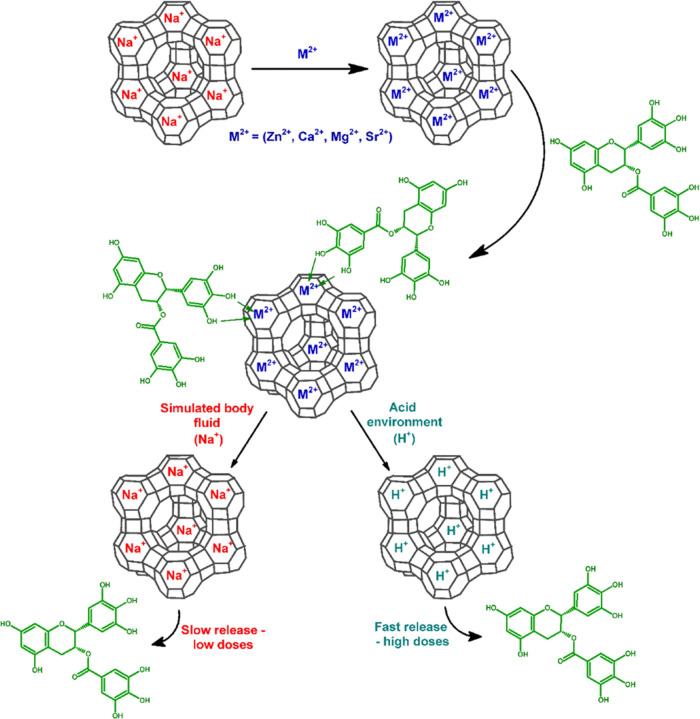
Scheme of research conducted in this work.

In this work, type X zeolites with Mg^2+^, Ca^2+^, Sr^2+^, and Zn^2+^ ions were prepared and used as epigallocatechin gallate carriers. Obtained ion-exchanged zeolites were characterized with various techniques to confirm successful ion exchange and drug sorption. The sorption capacity and release of the drug were examined for all of the prepared materials. The drug release profile under neutral and acidic conditions was determined for the materials. The scheme of the research carried out in this work is presented in Figure 1.

## 2 Materials and Methods

### 2.1 Materials

Zeolite NaX, zinc chloride, magnesium chloride, calcium chloride, strontium chloride, epigallocatechin gallate (EGCG), tris (hydroxymethyl) aminomethane (TRIS), sodium chloride, sodium bicarbonate, sodium sulfate, potassium phosphate dibasic trihydrate, potassium chloride, hydrochloric acid, bovine serum albumin (BSA), phosphate-buffered saline (PBS), acetic acid, and sodium acetate were purchased from Sigma-Aldrich

### 2.2 Ion Exchange

In order to obtain zeolites with divalent ions in the pores, ion exchange was performed. It was carried out by mixing, respectively, 40 mL of a solution of magnesium chloride, calcium chloride, strontium chloride, and zinc chloride at a concentration of 0.5 mol/dm^3^ with 2 g of commercial NaX zeolite. The samples were then placed on the rotator (speed 50 rpm) and left to mix for 24 h. After this time, the samples were centrifuged (speed 8000 rpm) and the whole process was repeated three times. In the next step, the obtained zeolites were washed three times with demineralized water to remove excess chlorides and then dried for 24 h at 100 °C.

As a result of ion exchange, the following materials were obtained: magnesium zeolite (MgX), calcium zeolite (CaX), strontium zeolite (SrX), and zinc zeolite (ZnX).

The described ion exchange procedure has already been used by the authors of this work in other publications, in which it was proved that ion exchange does not change the crystalline properties of the zeolite.^33,34^

### 2.3 EGCG Sorption

Sorption of EGCG was carried out by weighing 30 mg of MgX, CaX, SrX, and ZnX zeolites and then placing them in 15 mL tubes. 14 mL of the prepared EGCG solution in Tris–HCl buffer (pH = 7.4) at a concentration of 0.1 mg/mL was poured into each sample. In the next step, the tubes were placed on the rotator and mixed (speed 50 rpm) for 24 h at room temperature. Subsequently, all samples were centrifuged for 10 min (speed 4500 rpm), after which 1 mL of the solution was taken and placed in a UV cuvette to measure their absorbance by UV–vis spectroscopy. After the analysis, the collected solution samples were placed back in the appropriate tubes, which were then shaken to mix the zeolites with the drug. Samples prepared in this way were placed on a rotary stirrer for 24 h, and all steps were performed again. The changing concentration of EGCG in the solutions was examined after 1, 2, and 3 days.

As a result of sorption, the following materials were obtained: MgX-EGCG, CaX-EGCG, SrX-EGCG, and ZnX-EGCG.

### 2.4 Drug Release in Simulated Body Fluids

After EGCG sorption, desorption was performed to determine the time and profile of drug release. 2 mL of simulated body fluids at pH 7.4 was added to the drug-attached zeolites.^26^ The samples were placed on the rotator and mixed (speed 50 rpm) for 1 h, then centrifuged (10 min, speed 4500 rpm), and the amount of drug released was analyzed by UV–vis spectroscopy. The material was then flooded with a new batch of SBF to supply the sodium ions, which removes the divalent ions present in the pores of the zeolites. After another hour, drug release was tested again, and the interval between testing was increased to 24 h, then 3 days, and then to 7 days, due to the low amounts of EGCG released.

### 2.5 Drug Release under Acidic Conditions

Zeolite samples after sorption were also subjected to separate desorption in an acidic environment. For this purpose, 2 mL of an acetate buffer solution of pH 5 was added to the zeolites with EGCG attached.^35^ The materials were placed on a rotator (speed 50 rpm) and stirred for 15 min, then centrifuged (10 min, speed 4500 rpm), and examined with UV–vis spectroscopy to determine the amount of released epigallocatechin gallate. In the next step, the samples were flooded with a new portion of acetate buffer, and after 15 min, another UV–vis spectroscopy test was performed.

### 2.6 Drug Release under Mixed Conditions

The interaction of EGCG with the zeolite and the resulting bonds may be strong enough to protect the compound from degradation in healthy tissues. To test whether the drug under simulated body fluid conditions is protected by the zeolites from being released in large amounts, the samples initially desorbed in SBF were then placed in an acidic environment. For this purpose, after desorption in SBF, the zeolites were flooded with 2 mL of an acetate buffer solution at pH 5, placed on a rotator (speed 50 rpm), and centrifuged after 10 min (10 min, speed 4500 rpm), and then the amount of released EGCG was checked using UV–vis spectroscopy.

### 2.7 BSA Protein Adsorption Test

The BSA protein adsorption test was performed to determine the bioavailability and biocompatibility of the materials used, and it was carried out on the basis of the procedure described in the publication.^36^ For this purpose, a bovine serum albumin (BSA) solution was prepared by dissolving 400 mg of BSA in 16 mL of phosphate-buffered saline (PBS). 10 mg of MgX-EGCG, CaX-EGCG, SrX-EGCG, and ZnX-EGCG was placed in Eppendorf tubes and flooded with 1.5 mL of BSA solution. The samples were placed on a rotator and mixed for 3 days. The materials were then centrifuged to recover the solids, rinsed three times with demineralized water, and allowed to dry.

The following materials were obtained: MgX-EGCG-BSA, CaX-EGCG-BSA, SrX- EGCG-BSA, ZnX-EGCG-BSA.

### 2.8 Methods

#### 2.8.1 Scanning Electron Microscopy (SEM) and Energy-Dispersive Spectroscopy (EDS)

SEM images were recorded with the use of a scanning electron microscope VEGA 3 (TESCAN, Czech Republik). The SEM toll was equipped with an EDS analyzer (Bruker, U.K.). EDS was used to conduct elemental analysis of the samples. Two magnifications were used: 5 and 20 kx.

#### 2.8.2 Transmission Electron Microscopy

Tested materials were scattered into copper mesh coated with carbon film and analyzed with a transmission electron microscopy (TEM) HT7700 (Hitachi) at 100 kV of accelerating voltage.

#### 2.8.3 Fourier Transform Infrared Spectroscopy

FT-IR analysis of all materials was performed by using a Vertex70 spectrometer (Bruker Optics, Germany). All materials were studied by using a single reflection diamond ATR crystal. The tests were carried out in the spectral range of 4000–600 cm^–1^ with a resolution of 4 cm^–1^ and 32 scans for signal accumulation.

#### 2.8.4 UV–Vis Spectroscopy

A UV–vis–UV 2600 spectrophotometer (Shimadzu, Japan) was used to determine changes in the concentration of epigallocatechin gallate in the process of sorption on zeolites and during release under SBF and acidic conditions. The measurements were carried out at a wavelength in the range of 240–350 nm with a maximum at 272.5 nm. During sorption, a Tris–HCl solution was used as a background, while during desorption, a solution of simulated body fluids and acetate buffer was used, depending on the process conditions. The amount of drug retained on the zeolite was calculated from the EGCG calibration curve in 0.1 M Tris–HCl solution at pH 7.4, and the amount of drug released was calculated from the EGCG calibration curve in SBF or acetate buffer at pH 5.

## 3 Results

**Figure 2:**
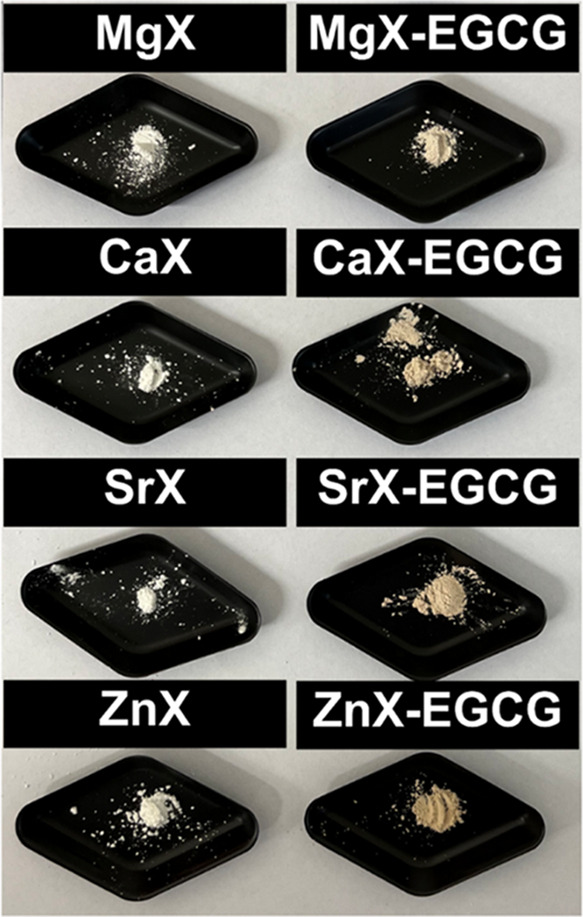
Photograph of samples before and after EGCG sorption.

Photos of the powders analyzed in this work are presented in Figure 2. As can be seen, the changes after the sorption of the drug are visible to the human eye. The zeolite used is white, while the powders after sorption of EGCG change color to a darker one. It is also noticeable that ZnX-EGCG is the darkest, and MgX-EGCG is the lightest. The color change is due to the fact that the EGCG complexes with ions are colored, which has been previously described in other works.^37^

**Figure 3:**
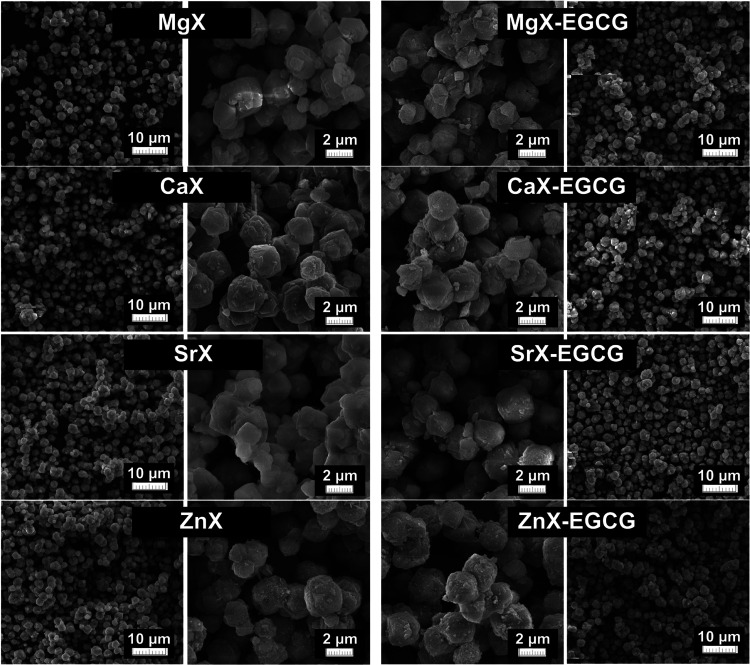
SEM images before and after EGCG sorption.

The samples were also characterized by using SEM analysis, which shows the surfaces and morphologies of potential carriers. SEM images before and after drug sorption are presented in Figure 3. Analyzing the SEM images of the MgX, CaX, SrX, and ZnX zeolites, it can be seen that the morphology of the materials shows a homogeneous distribution of small particles (2–3 μm) with a shape characteristic of the X-type zeolite. This means that the inclusion of magnesium, calcium, strontium, and zinc ions does not change the appearance and size of the zeolite particles, and the elements did not deposit on the surface of the materials in the form of chlorides or oxides. Moreover, it can be seen that the ion exchange does not affect the agglomeration of the particles, which could eliminate them as potential drug carriers.^34,35^ In the case of SEM images after the epigallocatechin gallate sorption process, the zeolites do not differ significantly from their images before drug attachment. The particles are typical of a microporous aluminosilicate structure with regularly occurring crystals. This indicates that the drug has not precipitated on the surface of the materials and probably binds to the carriers through ions located inside the pores and on the surface of the zeolites through coordination bonds. The drug layer is so thin that it is not visible in the SEM images. The lack of additional structures and the formation of only a thin layer indicate that the drug will most likely be released to a similar extent from each dose of the carrier.

**Figure 4:**
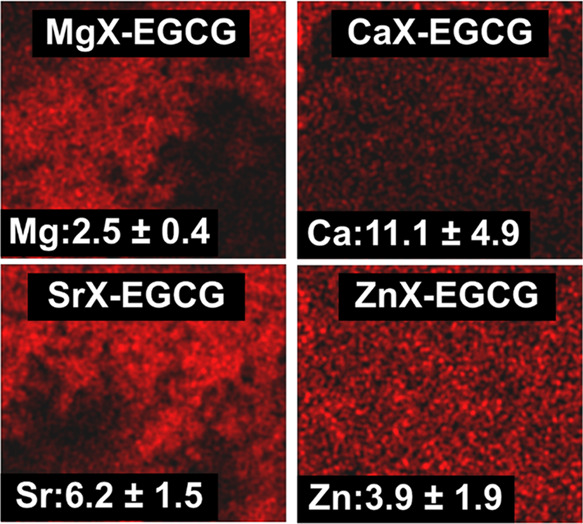
Distribution and content of divalent ions in the prepared materials.

The important information is how the ions are distributed after drug sorption. The distribution of magnesium, calcium, strontium, and zinc ions is shown in Figure 4. Divalent ions in all materials are evenly distributed. This is important because the presence of ion clusters would indicate that they are eluted and separate ion-EGCG complexes not bounded to the carrier are formed. This phenomenon does not occur here, which also indicates that the drug must be evenly distributed. The material with the most amount of divalent ions is CaX-EGCG and the least MgX-EGCG.

**Figure 5:**
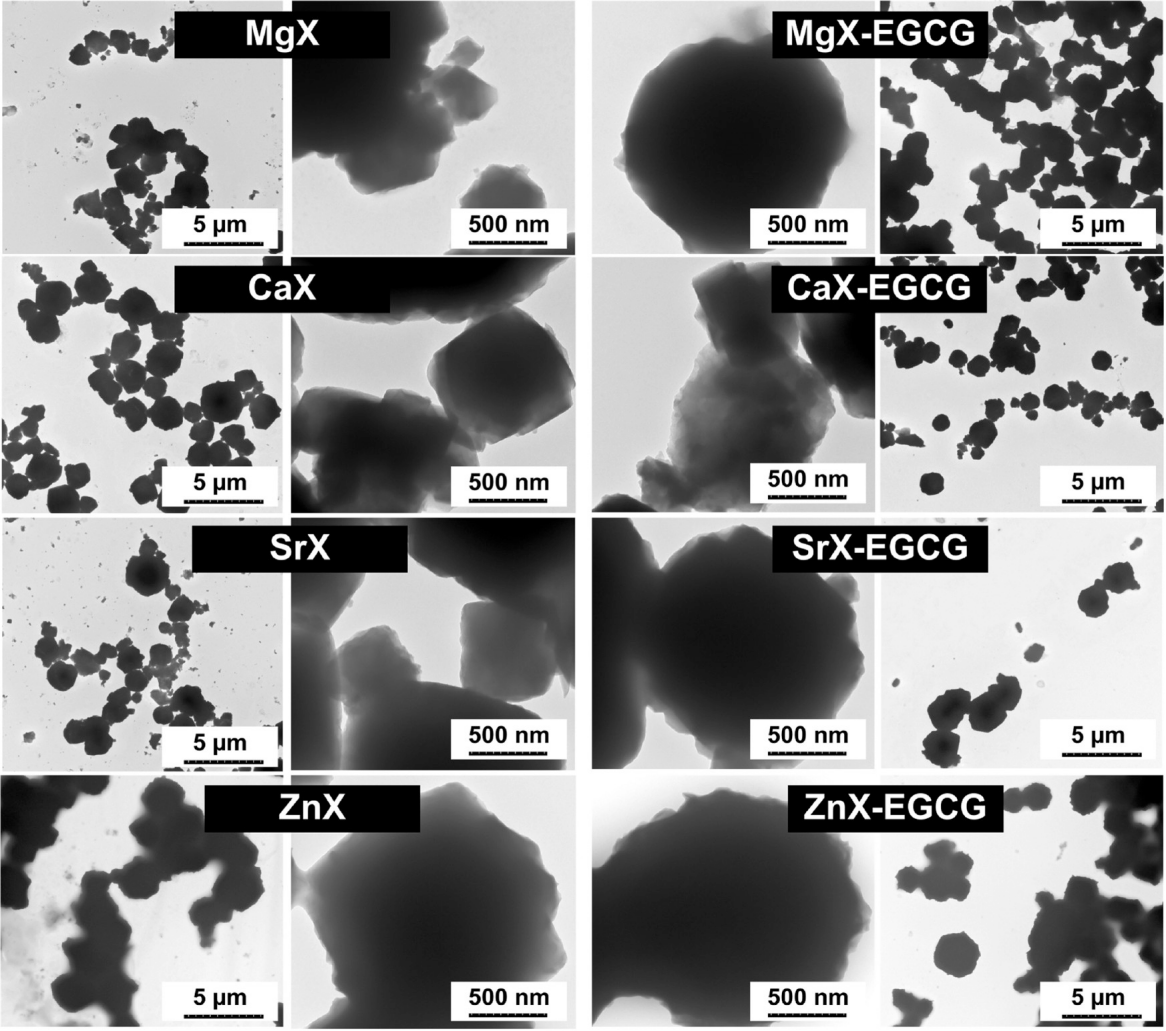
TEM images before and after EGCG sorption.

The materials were additionally characterized by using TEM analysis (Figure 5). As in the SEM analysis, zeolite particles with a size of 2–3 μm are usually visible. Zeolites after ion exchange, regardless of the cation, do not differ significantly from each other and no precipitations are visible, which proves that the ions of magnesium, calcium, strontium, and zinc were incorporated only on the basis of ion exchange, and not, for example, precipitation in the form of oxides or salt on the surface. After sorption of the drug, there are no visible new structures that could indicate the precipitation of the drug outside the surface of the zeolite. The edges of the zeolites observed at higher magnification do not differ significantly from each other. The lack of significant changes indicates that EGCG is most likely complexed as a thin layer on the surface of the carriers.

**Figure 6:**
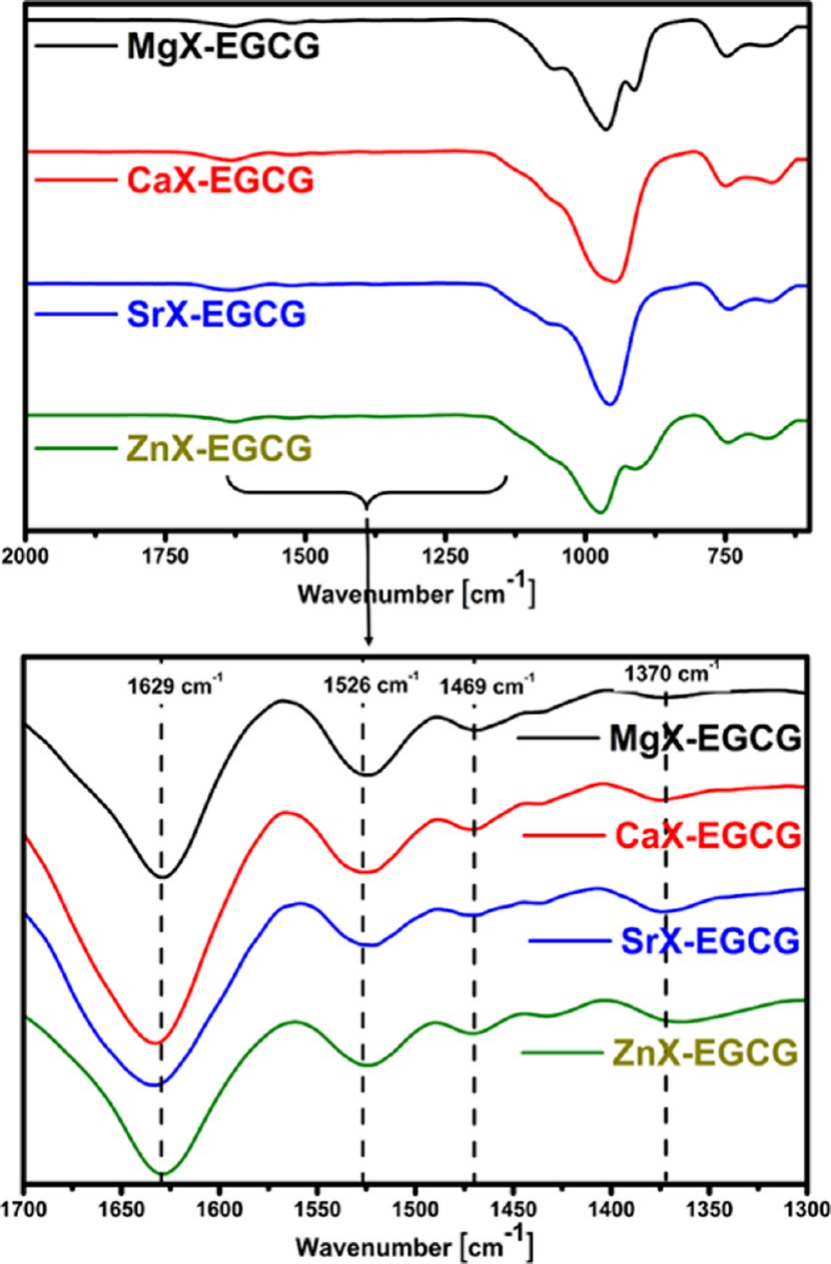
FT-IR spectra of zeolites after the sorption of EGCG.

The use of Fourier transform infrared spectroscopy was aimed at confirming the effectiveness of EGCG sorption on zeolites by identifying the appropriate functional groups. Spectra of zeolites after the sorption of EGCG are presented in Figure 6. Bands characteristic of aluminosilicates can be seen in the spectra. The bands in the range of 1100–600 cm^–1^ correspond to the vibrations of the aluminosilicate skeleton. In the spectrum of each of the tested materials, the most visible are wide bands of high intensity in the range of 1100–940 cm^–1^.^38^ They are attributed to internal, asymmetric stretching vibrations T–O of the TO_4_ tetrahedron, where T = Si or Al. In addition, two characteristic bands in the range of 780–600 cm^–1^ are also visible, which are assigned to symmetrical Al–O stretching vibrations in the Si–O–Al system.^39^ In order to show bands characteristic of EGCG, it was necessary to show only a fragment of the spectrum in greater detail (bottom, Figure 6). The obtained results show that EGCG was sorbed on all materials. The band at 1629 cm^–1^ is characteristic of the aromatic rings that occur in EGCG molecules.^40^ In addition, bands of low intensity are visible at 1526 and 1469 cm^–1^, corresponding to the stretching vibrations of C=C bonds in the aromatic ring.^41^ In the case of zinc, calcium, and strontium zeolite, a band around 1370 cm^–1^ is also visible, which can be attributed to the bending vibrations of the OH groups in the phenolic rings of EGCG.^40,41^

**Figure 7:**
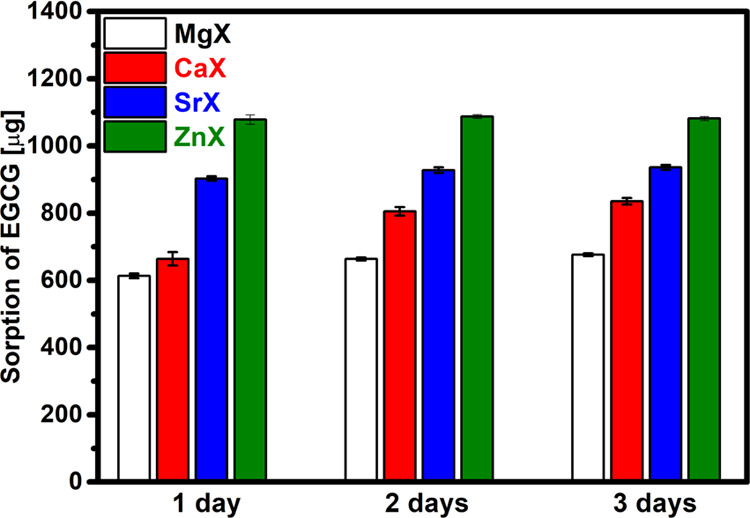
Amount of EGCG sorbed on the tested materials.

The results presented in previous studies indicate that the drug is retained but do not indicate how much it is. During the sorption of the drug, the amount that was successfully sorbed was tested by using UV–vis spectroscopy. Sorption of the drug on the modified zeolites was carried out for 3 days. Measurements using a UV–vis spectrometer were made 1, 2, and 3 days after flooding the samples with epigallocatechin gallate solution. The absorbance was measured to observe changes in the concentration of the EGCG solution and to determine the effectiveness of the process. The amount of drug retained on the magnesium, calcium, strontium, and zinc zeolite is shown in Figure 7. Based on the results, it can be concluded that the type of ions present in the pores of the zeolite affects the amount of drug retention. Despite flooding the samples with the same amount of EGCG solution, sorption on selected materials occurred to a different extent. The amount of drug retained was the highest for zinc zeolite and amounted to 1081.95 μg, which is about 79% of the total amount that could be attached. As can be seen from the results of the EDS analysis (Figure 4), this is the material that did not have the most divalent ions but nevertheless retained the most drug. The highest values for this zeolite most likely result from the properties of zinc and its ability to form strong complex compounds. In the case of other materials, these amounts were lower for strontium, calcium, and magnesium zeolite and amounted to 67%, 60%, and 48%, respectively. Greater sorption efficiency for ZnX, CaX, and SrX zeolites is also confirmed by the analysis of the FT-IR spectrum, which shows an additional band around 1370 cm^–1^, invisible for MgX (Figure 6). In the case of magnesium zeolite, the small amount of drug retained is most likely due to the low amount of magnesium ions, as indicated by the EDS analysis. Comparing individual days, it can be seen that increasing the time does not significantly affect the amount of EGCG retained.

**Figure 8:**
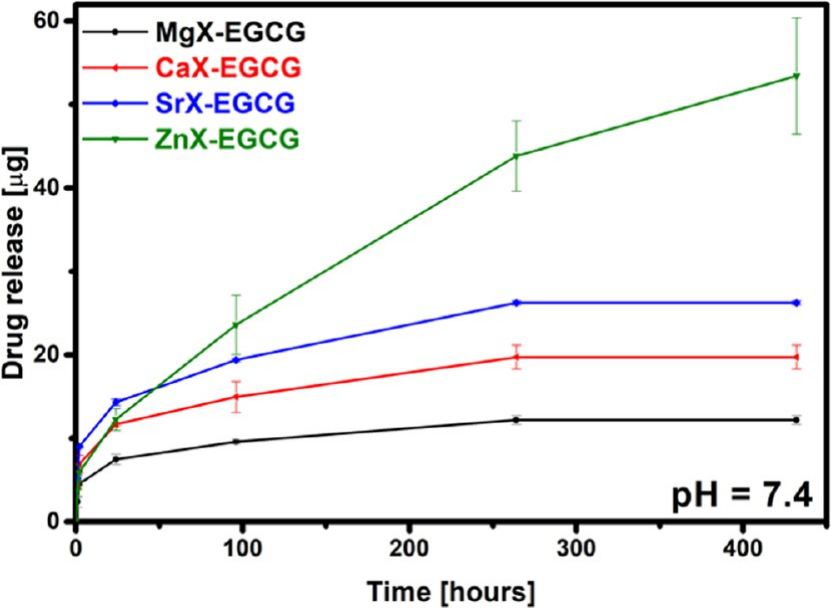
EGCG release in the SBF environment.

The most important stage of the research was to determine how much and when EGCG would be released from the potential zeolite carriers. Figure 8 shows the amount of EGCG released from zeolite carriers by SBF over 432 h (18 days) for all materials. The desorption of EGCG from individual zeolites occurs to a different extent depending on its modification. During the first 24 h, most of the drug is desorbed from zeolite modified with strontium ions, while after 96 h, the largest amount is observed for ZnX. Upon completion of the process, significantly more EGCG was released from the zinc zeolite than from the other materials. In this case, due to the greater amount of drug retained on the carrier, the amount of EGCG released is also correspondingly greater. In turn, the very small amount of substance released from MgX may be due to the small amount of magnesium ions present on the surface and in the pores of the material, which is confirmed by the EDS analysis. Such a small amount of the released substance in conditions resembling the environment of the human body indicates the potential of the materials used in the case of long-term, prolonged release. This prevents the secretion of too much of the active substance in too short a time, thanks to which the maximum safe concentration will not be exceeded, and at the same time it will not cause a toxic effect. In addition, the desorbing of the substance from the zeolites over a long period of time due to ion exchange indicates the potential for the use of the carriers in the treatment of osteoporosis (e.g., from zeolite-modified titanium implants^42^), where the delivery of small doses of the drug is desirable.

**Figure 9:**
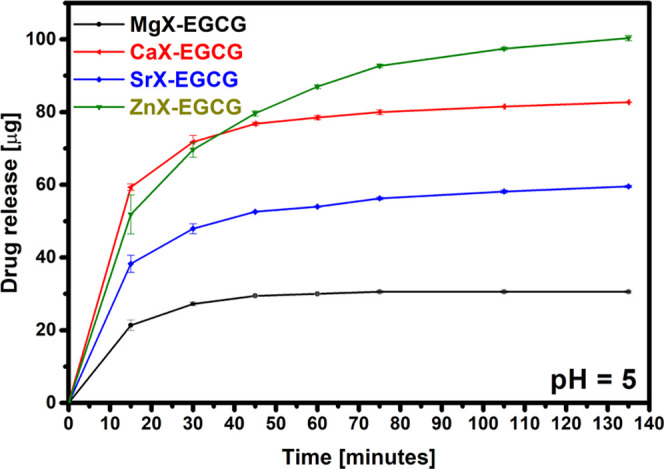
EGCG release in an acid environment.

The desorption of EGCG under acidic conditions (pH 5) proceeded in a different way than the release under the influence of simulated body fluids. As shown in Figure 9, the process took place in a shorter time and the amount of drug released was greater. The zeolite modified with calcium ions released the most drug during the first 15 min, but after 45 min, the highest amounts of desorbed EGCG are observed for the zinc zeolite. Together with the drug, ions of elements contained in the pores of zeolites are also released. In the case of zinc, the ubiquity of this element in many important biological processes suggests that its deficiency may be associated with the development and progression of cancer, therefore maintaining its appropriate concentration and supplying it with the drug may help in the treatment.^43^ The release of much larger amounts of EGCG from zeolites in a shorter time in a slightly acidic environment indicates the potential of the materials to be used for drug delivery to cancerous tumors whose pH is more acidic than that of healthy tissues. Epigallocatechin gallate is targeted at cancer tissues without affecting healthy cells, which is an advantage over other drugs used in the treatment of cancer.^10^ Complexing with zeolite may also increase its effectiveness and plasma concentration, which after oral administration does not exceed 0.32%.^1^

**Figure 10:**
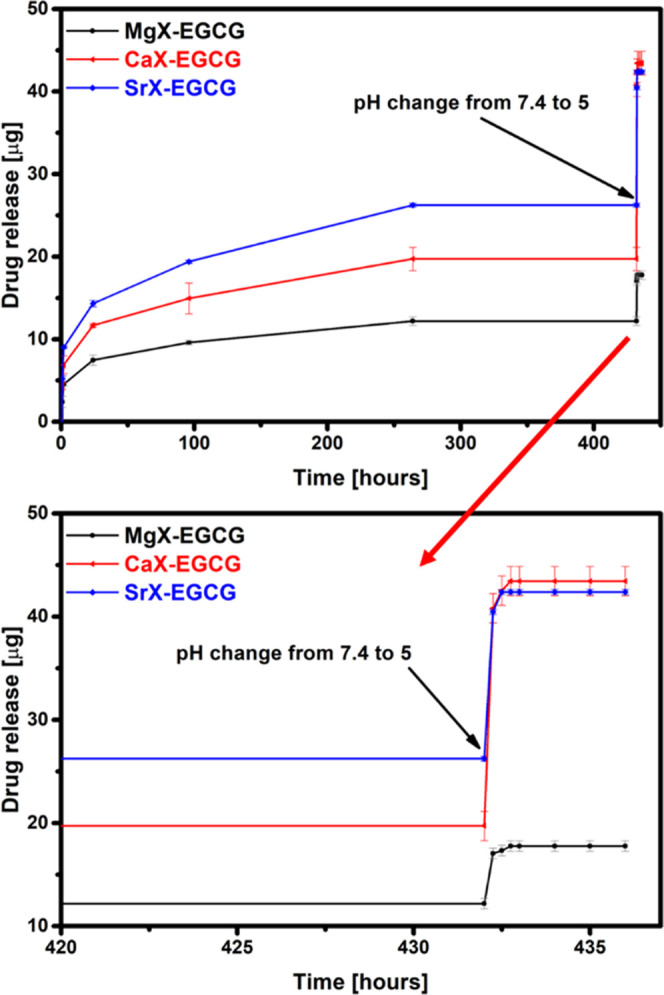
Release of EGCG in a mixed medium from magnesium, calcium, and strontium zeolites.

**Figure 11:**
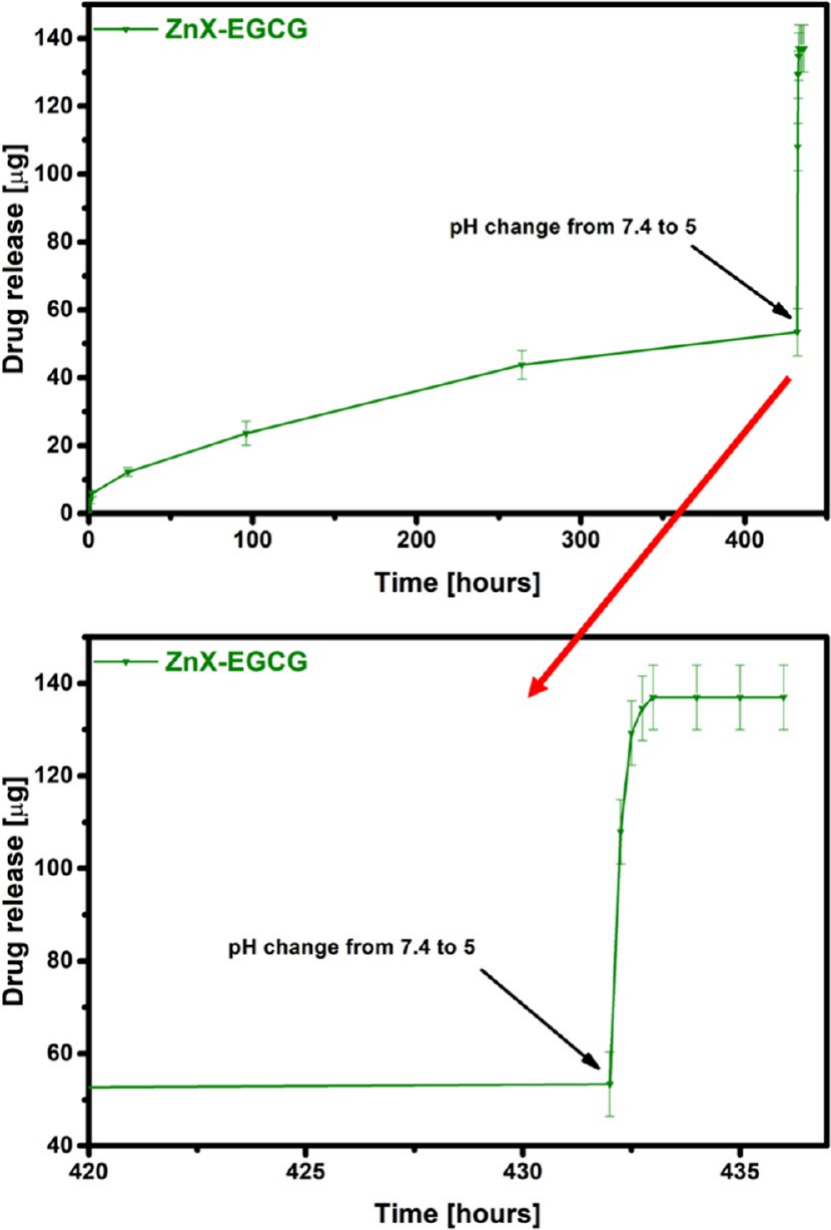
Release of EGCG in a mixed environment from a zinc zeolite.

The very small amounts of EGCG released from the zeolites in SBF may be due to the complexation of the drug with the carrier and at the same time its protection against degradation in other tissues. After placing the previously desorbed compounds in SBF in a slightly acidic environment (pH 5), the values of the released substance increased sharply for all materials (Figures 10 and 11). For each of the materials, after changing the pH, there was a large, single release of the drug, and then, this value oscillated at a similar level. The greatest difference in the amount of released EGCG is visible for the zinc zeolite. Much higher amounts of released EGCG when added to an acidic environment indicate that in a solution of simulated body fluids, the zeolite probably forms a stable complex with EGCG, from which the desorption process takes much longer than in the case of slightly acidic pH. It implies that the obtained carriers, and in particular ZnX, have the potential to release substances in response to a change in the acidity of the environment and can be used in controlled drug release systems. Therefore, these materials can protect the drug from degradation in healthy tissues of the body and rapidly release EGCG only when it reaches cancer cells, which makes them promising materials for use in cancer treatment.

**Figure 12:**
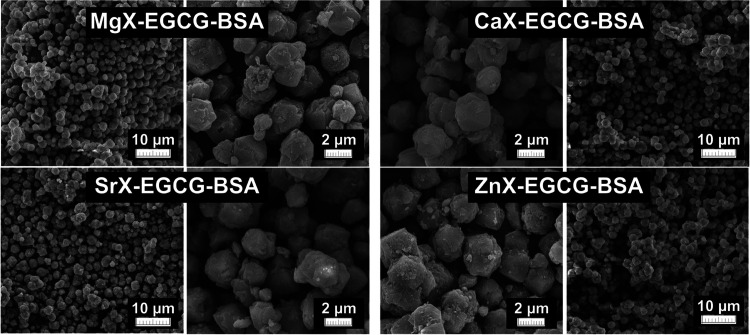
SEM images after BSA adsorption.

The biocompatibility of Zeolite X has already been proven in several studies. Lutzweiler et al. proved that magnesium and calcium zeolites do not have cytotoxic properties.^44^ Cytotoxicity studies were also carried out for the zinc zeolite and the absence of zeolite toxicity was proven using MCF-7 cells.^25^ In this work, the adsorption of proteins on zeolite carriers with retained drug was investigated. The adsorption of BSA, the most abundant protein in the blood, plays a positive role in the body’s response to foreign material and confirms the biocompatibility of the compounds used. Comparing the surface images of the compounds before (Figure 3) and after BSA modification (Figure 12), the particle size did not change after sorption in the case of zeolites. In addition, the particles do not agglomerate, and no precipitation is visible on their surface. These results do not confirm the presence of protein on the surface but may indicate the formation of a single layer of BSA. This makes the obtained materials promising for biological applications.^36,45^

**Figure 13:**
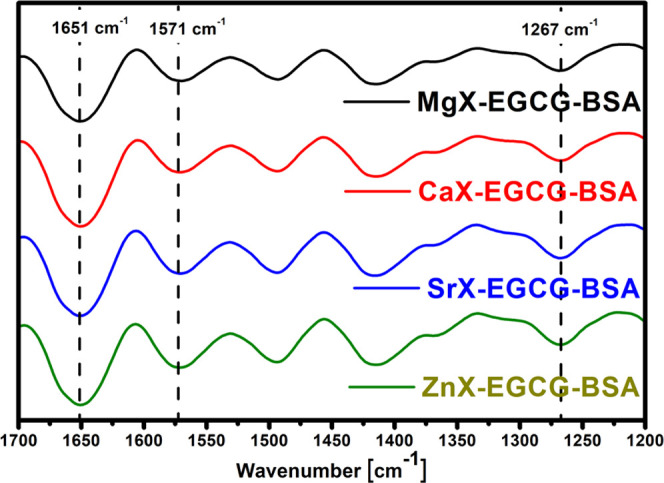
FT-IR spectra of zeolites after the sorption of EGCG and adsorption of BSA.

In order to confirm that the BSA layer is actually on the surface, FT-IR analysis was also performed (Figure 13). In the case of zeolite spectra with EGCG and BSA, new bands are visible, which confirms the effectiveness of BSA adsorption. All materials have bands at around 1651 cm^–1^. This band corresponds to C=O stretching vibrations in BSA peptide bonds. Also visible is a band near 1571 cm^–1^ that is attributed to the second amide band in BSA, corresponding to the C–N stretching vibration coupled to the N–H bending vibration. In addition, at a wavelength of about 1267 cm^–1^, a third amide band is also visible.

The material presented in this paper seems to be an interesting alternative to the previously described EGCG delivery systems (Table 1). It should be emphasized that zeolites have never been used for this application before. Materials that release EGCG slowly have been previously described in the literature. The advantage of the material described in this work over the others may be that at neutral pH, the drug is released from it in a controlled slow manner, while at acidic pH, there is a large release of the drug. This allows for controlled targeted drug release in the treatment of both osteoporosis and cancer.

**Table 1:** Other EGCG Delivery Systems Described in the Literature^51^

drug carrier type	neutral pH	acidic pH	ref
gold nanoparticles	fast		(46)
PLGA nanoparticles	fast		(47)
hydroxyapatite	slow	slow	(48)
chitosan hydrogel modified with lanthanum	slow		(49)
bovine serum albumin/pullulan nanoparticles	fast		(50)
layered EGCG/Montmorillonite hybrid	slow	slow	(52)
zeolite with divalent ions (this work)	slow	fast	this work

## 4 Conclusions

The results presented in this paper confirm the effectiveness of zeolite carriers in the controlled delivery of EGCG. The type of divalent ion incorporated into the zeolite structure has a great influence on the sorption and release of the drug. The least amount of EGCG is retained on magnesium zeolite and the most on zinc zeolite. The release of the drug in low doses in a neutral environment indicates the high potential of the materials in the treatment of osteoporosis, while the release of the drug in a high dose in an acidic environment indicates the possibility of use in the treatment of cancer. The results also show that zeolites “protect” EGCG in a way by complexing it on their surface. The zeolites described in this work, due to their biocompatibility and unique properties as carriers, which has been proven by research, are an interesting alternative to the materials described so far. To the best of our knowledge, there are no materials in the literature with properties similar to those presented in this work. The results presented in this paper are very promising, and further research should focus on using the knowledge gained in this work in research aimed at osteoporosis or cancer.

## Data Availability

All data generated or analyzed during this study are included in this published article.
